# Supplementary material for: Acidic Shift of Optimum pH of Bovine Serum Amine Oxidase upon Immobilization onto Nanostructured Ferric Tannates
Source: Int J Mol Sci. 2022 Oct 12;23(20):12172. doi: 10.3390/ijms232012172 (PMC9603626; doi:10.3390/ijms232012172)
Supplement: Supplementary file 1 [file ijms-23-12172-s001.zip › ijms-1954275-supplementary.pdf]

Acidic Shift of Optimum pH of Bovine Serum Amine Oxidase upon Immobilization onto Nanostructured Ferric Tannates

Graziano Rilievo[1,†], Alessandro Cecconello[1,†], Simone Molinari[2], Andrea Venerando[1], Lavinia Rutigliano[3,4], Gayathri T. Govardhan[1], Dinusha H. Kariyawasam[1], Ruth J. Arusei[1], Lucio Zennaro[5], Maria L. Di Paolo[5], Enzo Agostinelli[4,6], Fabio Vianello[1,6], Massimiliano Magro[1,*]

Supplementary Information

[1] Department of Comparative Biomedicine and Food Science, University of Padua, Viale dell'Università 16, 35020, Legnaro (PD), Italy;
[2] Department of Geosciences, University of Padua, Via Gradenigo 6, 35131 Padova, Italy;
[3] Department of Molecular Medicine, Sapienza University of Rome, Viale Regina Elena 291, 00161 Rome, Italy;
[4] Department of Sensory Organs, Sapienza University of Rome, Policlinico Umberto I, Viale del Policlinico 155, 00161, Rome, Italy;
[5] Department of Molecular Medicine, University of Padua, via Gabelli 63, 35121 Padova, Italy;
[6] International Polyamines Foundation 'ETS-ONLUS' Via del Forte Tiburtino 98, I-00159 Rome, Italy.

[*] Correspondence: massimiliano.magro@unipd.it; Tel.: 0039-049-8272638
[†] These authors contributed equally to this work

Theoretical background

1. Dependence of the catalytic constant of BSAO on pH

The bell-shaped dependence of k_{cat} values of BSAO on pH indicated the involvement of two ionizable species in the catalytic oxidation of the substrate. The pKa values of these groups, controlling the ascending and descending parts of the pH profile, were calculated according to the following equation [1]:

$$k_{cat} = k_{cat,0} \left(\frac{1 + \frac{\alpha K_{a1}}{[H^+]} + \frac{\beta [H^+]}{K_{a2}}}{1 + \frac{[H^+]}{K_{a2}} + \frac{K_{a1}}{[H^+]}} \right) \qquad (S1)$$

where $k_{cat,0}$ is the pH-independent catalytic constant, Ka_1 and Ka_2 are the acid dissociation constants of the two ionizable groups (1 and 2), and α and β are correction factors, accounting for the non-zero activity of the fully protonated and deprotonated enzyme-substrate intermediates.

In this view, the presence of two correction factors in the present equation (S1) represents a variation of the well-established Koudelka model [2] which actually contains a single adjustment parameter.

It deserves to be recalled that the latter was introduced by Koudelka in the original Tipton and Dixon equation for “a simplified reaction scheme” [3]. This being said, the modified version of the Koudelka paradigm reported here was chosen as it was successfully applied for describing the BSAO catalysis [1].

NMR relaxation rates of nuclear probes in the presence of a paramagnetic center

The nuclear relaxation times (T_1 and T_2) of nuclear probes in solution are influenced by the presence of paramagnetic species. Thus, the observed longitudinal and transversal relaxation rates (T_1^{-1} and T_2^{-1}) are: $T_{1obs}^{-1} = T_{1f}^{-1} + T_{1p}^{-1}$ and $T_{2obs}^{-1} = T_{2f}^{-1} + T_{2p}^{-1}$, respectively. Where T_{1f}^{-1} and T_{2f}^{-1} are the

relaxation rates of the ligand soluble in solution, and T_{1p}^{-1} and T_{2p}^{-1} are the relaxation rates of the nuclear probe due to the presence of the paramagnetic centers.

Generally, chemical exchange of the probe between the bulk solution and the coordination sphere of the paramagnetic center occurs according to Swift e Connick [4] where the corresponding nuclear relaxation rates (T_{1p}^{-1} and T_{2p}^{-1}) are:

$$\frac{1}{T_{1p}} = \frac{P_b q}{T_{1M}+\tau_m} \qquad \text{(S2)}$$

and

$$\frac{1}{T_{2p}} = \frac{P_b q}{T_{2M}+\tau_m} \qquad \text{(S3)}$$

where, P_b is the molar fraction of the bound ligand, q is the number of ligands coordinated to the paramagnetic center, τ_m is the residence time of the ligand to the paramagnetic center, and T_{1M} and T_{2M} are the relaxation times of the probe bound to the paramagnetic center in water solutions. These relaxation rates are described by Solomon-Bloembergen equations [5,6]:

$$\frac{1}{T_{1M}} = \frac{2}{15}\frac{\gamma_I^2 g^2 S(S+1)\beta^2}{r^6}\left(\frac{3\tau_c}{1+\omega_I^2\tau_c^2} + \frac{7\tau_c}{1+4\omega_S^2\tau_c^2}\right) + \frac{2}{3}S(S+1)\left(\frac{2\pi A}{h}\right)^2\left(\frac{\tau_e}{1+\omega_S^2\tau_e^2}\right) \qquad \text{(S4)}$$

and

$$\frac{1}{T_{2M}} = \frac{1}{15}\frac{\gamma_I^2 g^2 S(S+1)\beta^2}{r^6}\left(4\tau_c + \frac{3\tau_c}{1+\omega_I^2\tau_c^2} + \frac{13\tau_c}{1+4\omega_S^2\tau_c^2}\right) + \frac{1}{3}S(S+1)\left(\frac{2\pi A}{h}\right)^2\left(\tau_e + \frac{3\tau_e}{1+\omega_S^2\tau_e^2}\right) \qquad \text{(S5)}$$

Where ω_S and ω_I are the Larmor precession frequencies of the electron and of the nucleus, respectively, γ_I is the giromagnetic ratio, *g* indicates the Lande' g factor, β is the Bohr magneton, *S* the total electron spin, *r* the distance between the nucleus and the paramagnetic ion and the (2πA/h) factor represents the electron-nucleus hyperfine coupling constant in Hertz. The first term of both equations describes the dipole-dipole interaction between the electron spin, S, and the nuclear spin, I, which are characterized by a correlation time, τ_c. The second term arises from scalar interactions, which are characterized by a correlation time, τ_e [7]. The correlation times are defined as follows:

$$\frac{1}{\tau_c} = \frac{1}{\tau_M} + \frac{1}{\tau_R} + \frac{1}{\tau_S} \qquad \text{(S6)}$$

and

$$\frac{1}{\tau_e} = \frac{1}{\tau_M} + \frac{1}{\tau_S} \qquad \text{(S7)}$$

where τ_M is the residence time of the ligand into the first coordination sphere of the paramagnetic centre, τ_R is the correlation time of the rotational motion of the complex, τ_S is the relaxation time of the electron spin.

For Cu^{2+} complexes, such as active sites of copper containing enzymes, the term $\omega_S^2\tau_c^2$ is » 1 and the scalar contribution to the relaxation rates can be considered negligible [8]. Furthermore, type 2 macromolecular copper complexes, such as BSAO, are characterized by a τ_S value in the 1 to 5 x 10^{-9} s range [9]. Under these conditions, equations (S3) and (S4) can be reduce to:

$$\frac{1}{T_{1M}} = \frac{2}{15}\frac{\gamma_I^2 g^2 S(S+1)\beta^2}{r^6}\left(\frac{3\tau_c}{1+\omega_I^2\tau_c^2}\right) \qquad \text{(S8)}$$

and

$$\frac{1}{T_{2M}} = \frac{1}{15}\frac{\gamma_I^2 g^2 S(S+1)\beta^2}{r^6}\left(4\tau_c + \frac{3\tau_c}{1+\omega_I^2\tau_c^2}\right) + \frac{1}{3}S(S+1)\left(\frac{2\pi A}{h}\right)^2 \tau_e \qquad \text{(S9)}$$

Supplementary Results

The contribution of native BSAO to the longitudinal and transversal relaxation rates of a fluoride ion (T_{1p} and T_{2p}) were studied at different intensity of the main magnetic field (B_0) according to Solomon and Bloembergen [5,6]. Table 3 (main text) shows that the T_{1p}/T_{2p} ratio of $^{19}F^-$ was » 1 at all the pH values explored, and, at least at pH 7.0, a different effect of ionic strength was observed (Figure S1), indicating different contributions controlling longitudinal and transversal relaxation rates of $^{19}F^-$ in the presence of BSAO [7]. Moreover, the decrease of $\frac{T_{1p}^{-1}}{[BSAO]}$ of $^{19}F^-$ with increased magnetic field strength (from 0.42 to 7.4 T) suggests that the longitudinal relaxation rate was in the "fast exchange region", that is, T_{1p}^{-1} was mainly controlled by T_{1M}, see equation (S2). Additionally, the longitudinal

relaxation rate of $^{19}F^-$ was not influenced by ionic strength (Figure S1) and, from the measurements performed at different temperatures, an activation energy of 28.5 kJ mol^{-1} was calculated. Furthermore, measurements of $\frac{T_{1p}^{-1}}{[BSAO]}$ of $^{19}F^-$ as a function of fluoride ion concentration in the presence of BSAO allowed the calculation of the association constant of the F^--BSAO system, K_F, which resulted of 0.49 M^{-1} at pH 7.0 (see Materials and Methods), and permitted to calculate the (T_{1M} + τ_m) factor (see equation (S2)), which resulted to be 74 µs at 0.42 T, assuming that one fluoride ion binds to one BSAO molecule. Conversely, regarding the transversal relaxation rate of $^{19}F^-$, ($\frac{T_{2p}^{-1}}{[BSAO]}$), the independence on the main magnetic field (see Table 3 in the main text), the dependence on ionic strength (see Figure S1), and its positive activation energy (+23.6 KJ mol^{-1}), suggest that ($\frac{T_{2p}^{-1}}{[BSAO]}$) is controlled by a different physical phenomenon. In particular, T_{2p}^{-1} should be in the "slow exchange region", that is, it was controlled by τ_m (equation (S3)). This correlation time, characterizing the chemical exchange of F^- from the bulk solution to the active site of BSAO, resulted equal to 8 µs. This estimated value of τ_m allowed the calculation of T_{1M}, which resulted equal to 66 µs. Moreover, since τ_m » T_{2M}, it means that T_{2M} was dominated by the scalar contribution as found in the case of SOD (i.e., superoxide dismutase) [10]. In the hypothesis that $\omega_S^2\tau_e^2$ » 1 and $\omega_S^2\tau_e^2$ » 1 (i.e., a negligible scalar contribution to the relaxation rates, equation (S8)) an estimate of τ_c, the correlation time controlling T_{1M}, can be proposed according to Nowak et al. [11]: $\tau_c = 1 \times 10^{-9}$ s. From equation (S6), it appears that τ_c is determined by the shortest correlation time (i.e., the fastest process) and, since BSAO has a molecular mass of 180,000 Da, its rotational correlation time in water, τ_R, should not be shorter than 10^{-7} s. On the other hand, even the contribution of τ_M on τ_c should be negligible due to the lack of the effect of ionic strength on T_{1p}. Finally, the τ_c value should be controlled by τ_S, the correlation time of the electron spin relaxation of the paramagnetic copper ion. Typically, type 2 macromolecular copper complexes are generally characterized by τ_S values in

the range 1 to 5 × 10^{-9} s [9]. Using this value for τ_c, an estimated Cu^{2+}-F^- distance can be hypothesized [7], and at pH 7.0 resulted equal to 4.3 Å. According to this hypothesis, the phenomenon causingthe molar longitudinal relaxivity to increase upon pH lowering, could be due to modifications of the ligand field of the Cu(II), which cause a variation of τ_S. Differently, according to $\frac{T_{1p}^{-1}}{[BSAO]}$ values at pH 5.2 (Table 3, main text), the T_{1M} resulted equal to 32 μs. By using the already mentioned calculations, the τ_c value of the fluoride ion in the presence of native BSAO at pH 5.2 resulted to be 2 × 10^{-10} s and the distance between F^- and the copper ion of BSAO was estimated to be 3.1 Å.

Supplementary Figures

Figure S1. Binding isotherms of BSAO on SAMN@TA surface. Panels: A, the Giles model describing the saturation behavior of BSAO surface concentration (Q, mg BSAO g^{-1} SAMN@TA) against the concentration of soluble enzyme at the equilibrium (Ce, mg L^{-1}); B, the linearized form of the Langmuir model (equation reported in the main text) confirming the formation of a monomolecular layer. Measurements were carried out at constant SAMN@TA concentration (500 mg L^{-1}) as a function of BSAO concentrations in the 25-200 mg L^{-1} range.

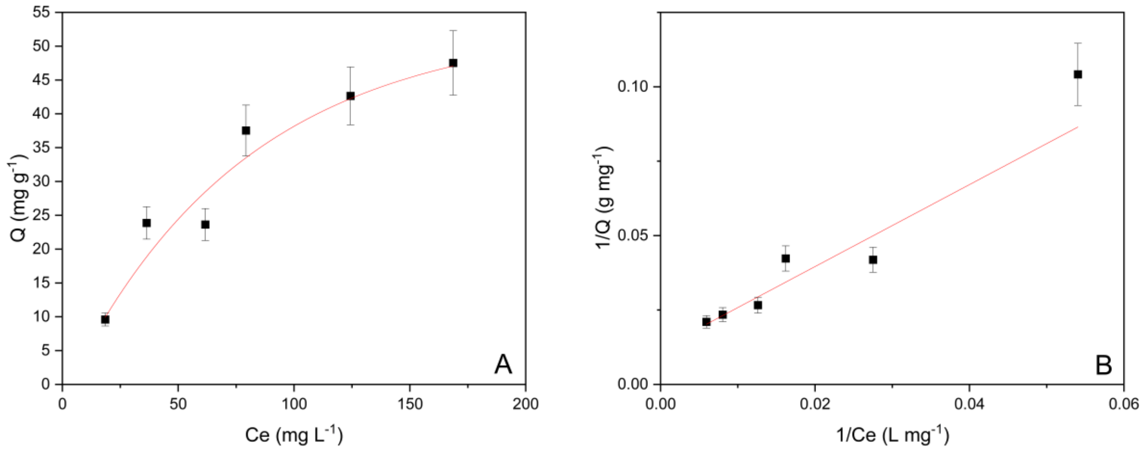

Figure S2. X-Ray diffraction images (a,c,e) of bare SAMNs, SAMN@TA and SAMN@TA@BSAO complexes and their corresponding diffractograms visible in b,d,f respectively. Mineral abbreviations: M=maghemite and H=hematite. Only the most intense peaks of the two phases have been marked. Maghemite (i.e., γ-Fe2O3) was identified as the major phase witnessing the integrity of the magnetic core both in SAMN@TA and SAMN@TA@BSAO. The increasing background, going form b to f, is related to the enhancement of an amorphous phase, very likely attributable to the single (SAMN@TA) and the double (SAMN@TA@BSAO) organic layers.

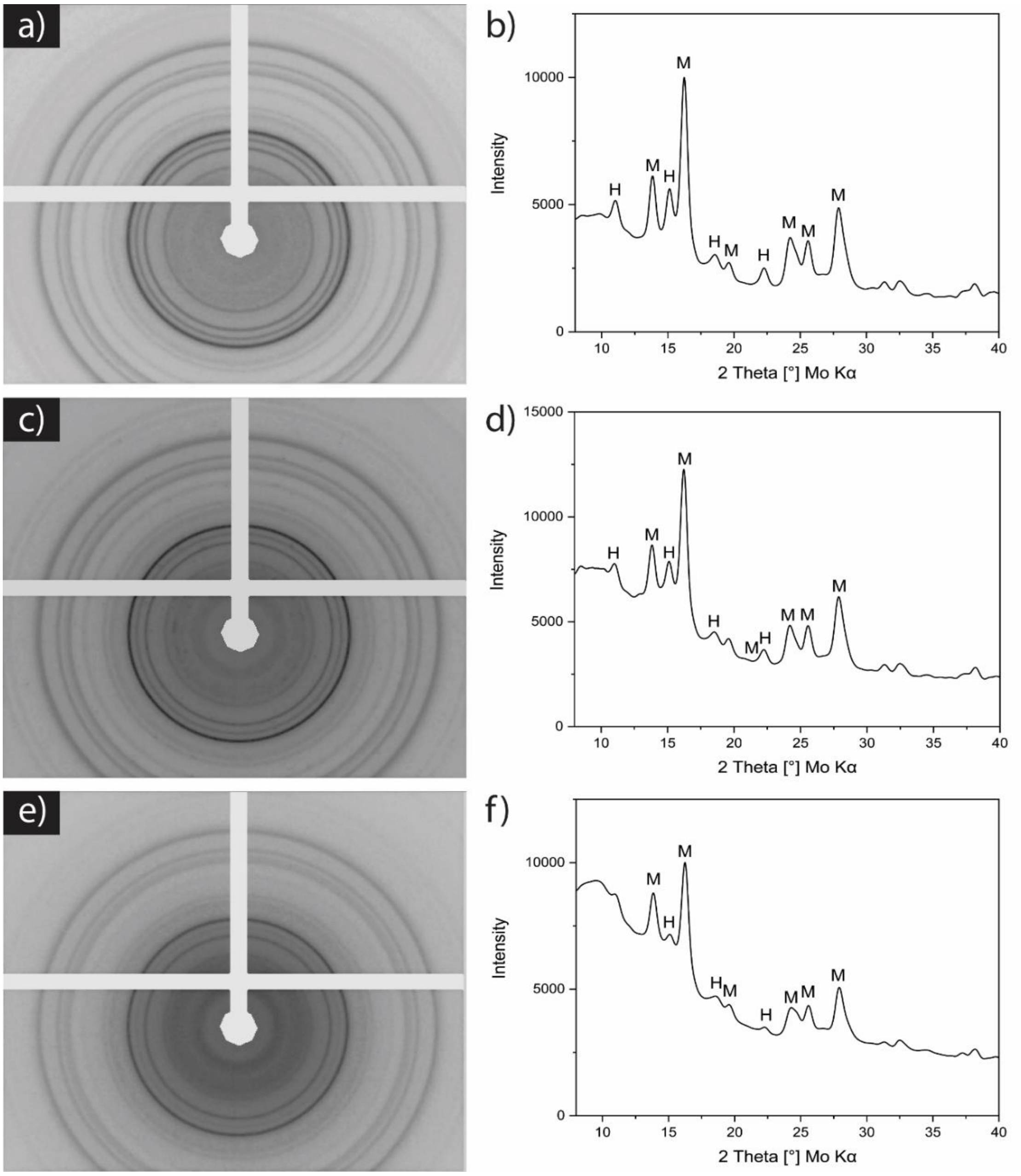

Figure S3. Scanning Electron Microscopy images of bare SAMNs (panel a), SAMN@TA (panel b), and SAMN@TA@BSAO (panel c) complexes. Energy Dispersive Spectroscopy spectra (panel d) of all investigated samples analyzed at 10 keV. Besides the presence of iron in all the samples related to the maghemite contribution, the carbon peak SAMN@TA and SAMN@TA@BSAO confirm the development of the carbonaceous shells.

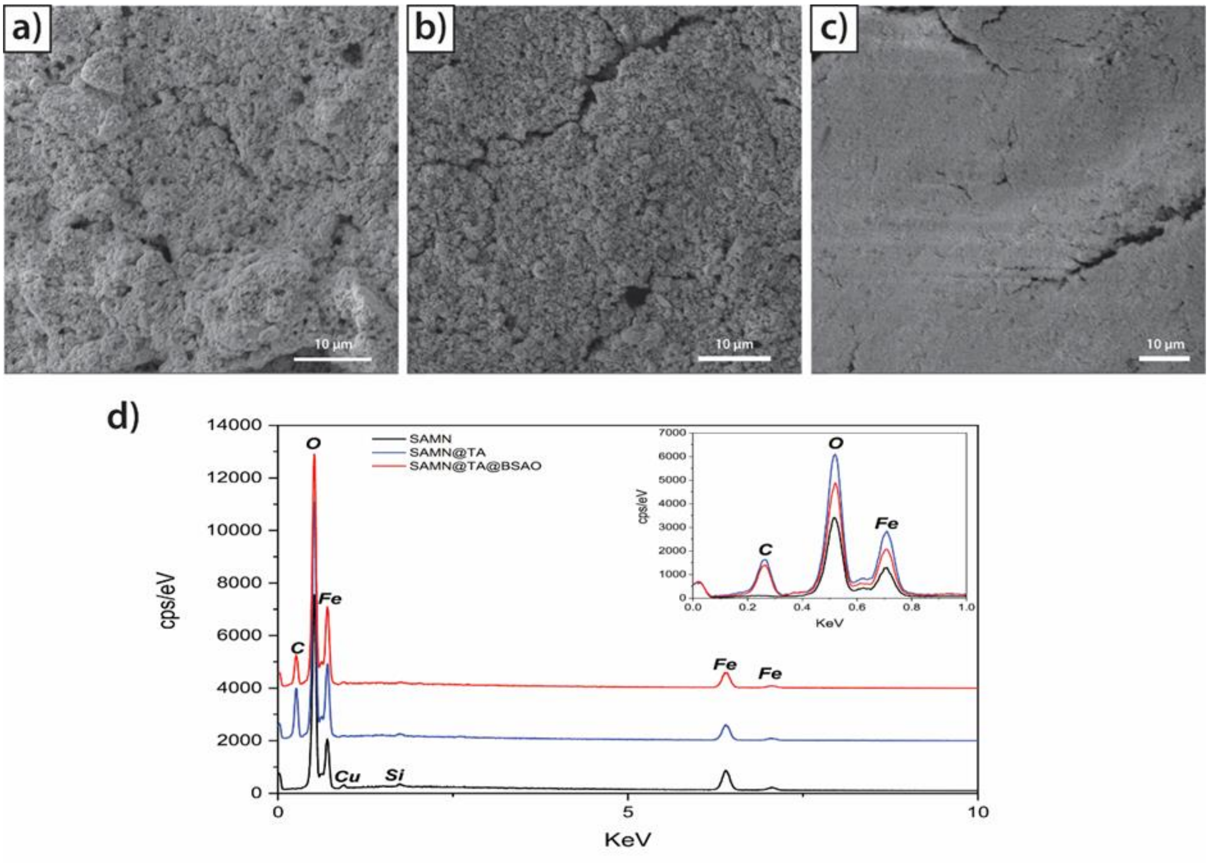

Figure S4. Michaelis constant (K_M) and catalytic efficiency, log (k_{cat}/K_M), of SAMN@TA@BSAO toward spermine oxidation at different pH values. A) Michaelis constant (K_M); B) Catalytic efficiency, log (k_{cat}/K_M)

A)

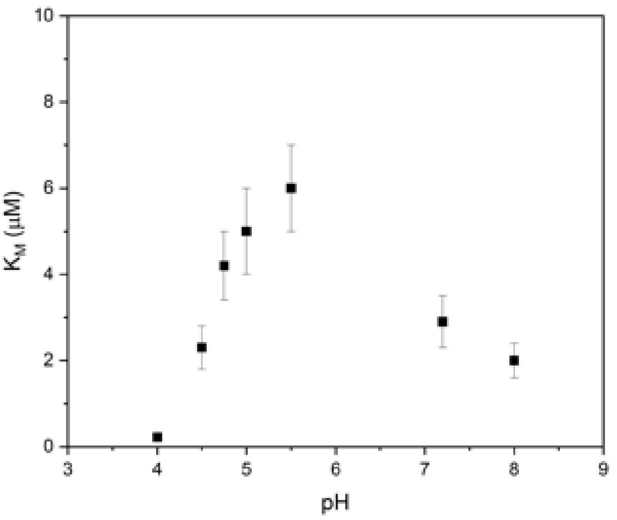

B)

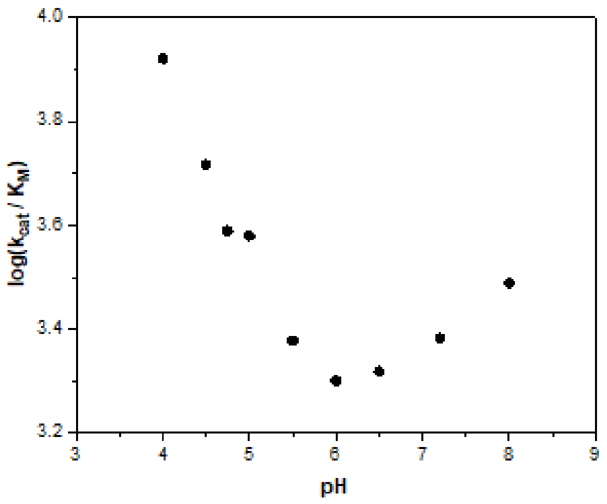

Figure S5. Dependence of the $^{19}F^{-}$ longitudinal (T_{1p}^{-1}/[BSAO]) and transversal (T_{2p}^{-1}/[BSAO]) molar relaxivity logarithms of native BSAO on the square root of the ionic strength, according to Debye-Huckel [12]. Measurements were performed at 7.4 T, T = 25°C, in 20 mM phosphate buffer at pH 7.0, containing 10 mM KF and 30 µM - 300 µM BSAO. Ionic strength was varied by adding small amount of 3.8 M KCl in 20 mM phosphate buffer, pH 7.0. (●) T_{1p}^{-1}/[BSAO]); (●) T_{2p}^{-1}/[BSAO].

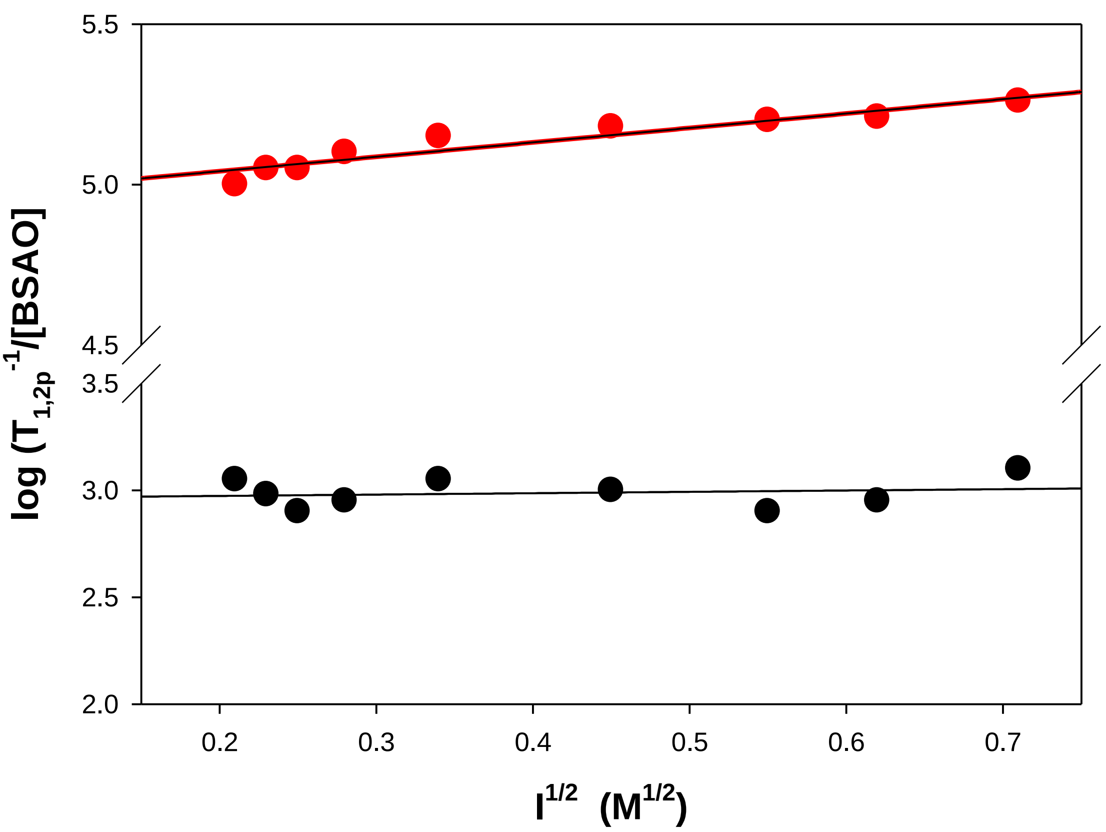

Figure S6. Michaelis-Menten curves of native 8.0 mg L^{-1} BSAO at pH 7.2 (a) and 50.0 mg L^{-1} SAMN@TA@BSAO at pH 5.0 (b) with spermine (SPM) as substrate.

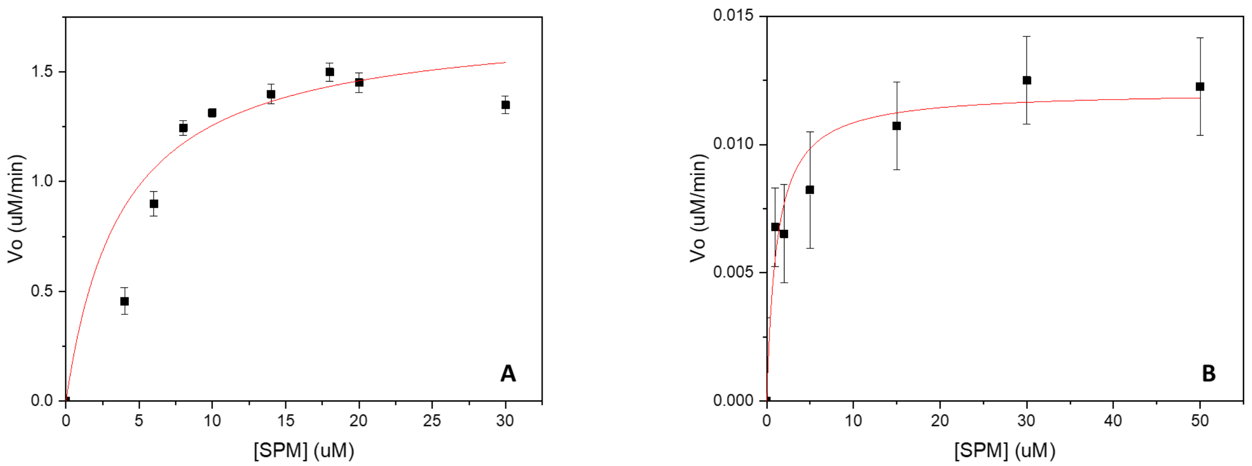

References

1. Di Paolo, M.L.; Vianello, F.; Stevanato, R.; Rigo, A. Kinetic Characterization of Soybean Seedling Amine Oxidase. *Arch. Biochem. Biophys.* **1995**, *323*, 329–334, doi:10.1006/ABBI.1995.9957.
2. Koudelka, G.B.; Hansen, F.B.; Ettinger, M.J. Solvent Isotope Effects and the PH Dependence of Laccase Activity under Steady-State Conditions. *J. Biol. Chem.* **1985**, *260*, 15561–15565, doi:10.1016/S0021-9258(17)36293-2.
3. Tipton, K.F.; Dixon, H.B.F. Effects of PH on Enzymes. *Methods Enzymol.* **1979**, *63*, 183–234, doi:10.1016/0076-6879(79)63011-2.
4. Swift, T.J.; Connick, R.E. NMR-Relaxation Mechanisms of O17 in Aqueous Solutions of Paramagnetic Cations and the Lifetime of Water Molecules in the First Coordination Sphere. *J. Chem. Phys.* **2004**, *37*, 307, doi:10.1063/1.1701321.
5. Bloembergen, N.; Purcell, E.M.; Pound, R. V Relaxation Effects in Nuclear Magnetic Resonance Absorption. *Phys. Rev.* **1948**, *73*, 712, doi:10.1103/PhysRev.73.679.
6. Solomon, I. Relaxation Processes in a System of Two Spins. *Phys. Rev.* **1955**, *99*, 559, doi:10.1103/PhysRev.99.559.
7. Dwek, R.A. *Nuclear Magnetic Resonance (N.M.R.) in Biochemistry : Applications to Enzyme*

Systems; Clarendon Press, Oxford, 1973; ISBN 0198546149.
8. Mildvan, A.S.; Cohn, M. Aspects of Enzyme Mechanisms Studied by Nuclear Spin Relaxation Induced by Paramagnetic Probes. *Adv. Enzymol. Relat. Areas Mol. Biol.* **1970**, *33*, 1–70, doi:10.1002/9780470122785.CH1.
9. Bertini, I.; Luchinat, C. *NMR of Paramagnetic Molecules in Biological Systems*; Benjamin/Cummings Pub. Co.,: Menlo Park, CA, USA, 1986; ISBN 080530780X.
10. Viglino, P.; Rigo, A.; Stevanato, R.; Ranieri, G.A.; Rotilio, G.; Calabrese, L. The Binding of Fluoride Ion to Bovine Cuprozinc Superoxide Dismutase as Studied by 19F Magnetic Relaxation. *J. Magn. Reson.* **1979**, *34*, 265–274, doi:10.1016/0022-2364(79)90002-7.
11. Nowak, T.; Maurer, P.J. Fluoride Inhibition of Yeast Enolase 2. Structural and Kinetic Properties of the Ligand Complexes Determined by Nuclear Relaxation Rate Studies. *Biochemistry* **1981**, *20*, 6901–6911, doi:10.1021/bi00527a025.
12. Scherer, P.; Fischer, S.F. Debye–Hückel Theory. In *Theoretical Molecular Biophysics*; Springer, Berlin, Heidelberg, 2010; pp. 45–59.
